# Hypnotherapy modulating early and late event-related potentials components of face processing in social anxiety

**DOI:** 10.3389/fpsyt.2024.1449946

**Published:** 2024-10-16

**Authors:** Han Zhang, Xinrong Xue, Jialin Wen, Yongyi Li, Chuan Fan, Lijun Ma, Huixue Wang, Mi Zhang, Shuyu Zhang, Die Hu, Kai Wang, Xiaoming Li

**Affiliations:** ^1^ Department of Medical Psychology, School of Mental Health and Psychological Science, Anhui Medical University, Hefei, China; ^2^ Department of Psychiatry, Chaohu Hospital of Anhui Medical University, Hefei, Anhui, China; ^3^ Department of Psychiatry, the First Affiliated Hospital of Anhui Medical University, Hefei, China; ^4^ School of Psychology, Australian National University, Canberra, Australia; ^5^ Institute of Artificial Intelligence, Hefei Comprehensive National Science Center, Hefei, China; ^6^ Anhui Province Key Laboratory of Cognition and Neuropsychiatric Disorders, Hefei, China; ^7^ Collaborative Innovation Center of Neuropsychiatric Disorders and Mental Health, Hefei, China; ^8^ Anhui Provincial Institute of Translational Medicine, Anhui Medical University, Hefei, China

**Keywords:** hypnotherapy, attention bias, social anxiety, face processing, event-related potentials (ERP)

## Abstract

**Background:**

Hypnotherapy has a potential role in modulating attention bias to treat social anxiety disorder (SAD). This study aimed to verify whether hypnotherapy can reduce social anxiety by changing attentional bias. The primary objective of our study is to explore the influence of hypnosis on various aspects of attention processes, specifically focusing on how it affects attention bias and social anxiety.

**Methods:**

This study included 69 participants with SAD who were assigned to three groups based on their scores on the Liebowitz Social Anxiety Scale (LSAS). The experimental group (n = 23) received a hypnosis treatment once a week, for a total of six sessions, while the control group (n = 23) and the baseline group (n = 23) did not receive any treatment. To evaluate whether hypnosis could alleviate SAD and attention bias towards threatening stimuli, we employed questionnaires and an odd-one-out task accompanied by electroencephalography (EEG) recordings.

**Results:**

Under the attention sensitivity conditions, the experimental group exhibited a reduced N170 and LPP at the posttest stage, and a similar N170 and LPP reduction under the attention disengagement conditions. Notably, the symptom improvements were positively correlated with the reduction in N170 and LPP amplitude across conditions.

**Conclusion:**

Hypnosis treatment modulates the early face processing and late emotional evaluation of threat-related stimuli in SAD patients. These findings suggest that N170 and LPP are important biomarkers for the treatment of SAD.

## 1 Introduction

Social anxiety disorder (SAD) is a common anxiety disorder typically characterized by a persistent state of fear when interacting with people, acting in front of people, or being observed or evaluated (1, 2). According to the DSM-5 description, people with SAD have uncontrollable and persistent fears. They feel afraid and uncomfortable when socializing with others, and some even have serious problems talking to others (3). SAD is one of the most prevalent and difficult-to-treat mental disorders (4). Epidemiological surveys in Western countries have shown that the lifetime prevalence rates of social anxiety are 10%-13%, and surveys of college students in the United Kingdom and the United States show that 10%-42% of college students have social difficulties or avoidance behaviors (5).

There is a growing body of literature that recognizes information processing biases play a vital role in SAD (6, 7). Attention bias, which has been highly correlated with SAD in numerous studies, has been the most explored part of all studies (8, 9). Furthermore, researchers have attempted to prove that SAD populations have a preference for processing negative emotional stimuli, and this attentional bias may have a causal effect on the formation and maintenance of anxiety (10–12). Regarding the specific features of attention bias, studies using the dot-detection paradigm showed that subjects with social anxiety responded more quickly to threat word locations compared to neutral word locations (13, 14). The cue-target paradigm examines the attentional bias towards threatening stimuli (15). The study showed a delayed shift towards emotional faces. The aforementioned evidence implies that individuals with SAD may exhibit enhanced processing of threats and struggle to divert their attention away from such threats.

Currently, the prevalent nonpharmacologic treatments for SAD are social skills training (SST), exposure *in vivo* (EXP), cognitive therapy (CT), and cognitive-behavioral therapy (CBT), which is the first line of treatment for SAD (16). In addition, a growing body of research uses attention bias modification (ABM) as a method to relieve social anxiety symptoms (17, 18). Hypnosis is a psychological technique in which the participant receives suggestions from the hypnotist and makes responses accompanied by imaginary experiences such as changes in perception, memory, and autonomous behavior (19). As a result, hypnosis can cause consciousness and cognitive changes after hypnotic suggestion (20). Some patients may exhibit increased tolerance to exposure therapy when employing hypnosis to facilitate imaginative exposure through vivid imagery and enhance relaxation (21). Raz et al. utilized post-hypnotic suggestions to successfully suppress the Stroop interfere effect in participants with high hypnotic susceptibility (22, 23). In an Event-Related Potential (ERP) investigation of the Simon effect, highly hypnotizable subjects performed decreased reaction times and increased accuracy with the hypnotic suggestion that noise stimuli were imperceptible. Concurrently, the latency of the P300 component during their responses to noise-incompatible trials showed a significant reduction (24). In one of our most recent studies, we discovered that hypnosis helps reduce test anxiety by altering students’ attentional bias through hypnotic suggestion (25). These findings suggest that hypnosis can reduce social anxiety symptoms and modify attentional bias.

The main objective of this research is to investigate whether hypnosis therapy can alleviate social anxiety by altering attentional bias. Additionally, we seek to ascertain which alterations in these attentional mechanisms confer benefits to individuals with SAD. To address these questions, we will investigate changes in social anxiety symptoms and attention bias before and after hypnosis using ERPs technology. ERPs exhibit a high temporal resolution, enabling then to discriminate between early and late attention processes when processing facial stimuli.

When studying the early processing of facial emotional expressions, one ERP component that must be mentioned is N170 (26), a face-sensitive ERP component (27). In particular, the N170 indicates efficient categorization of personally significant faces (28). The N170 amplitude gradually increases with the intensity of emotional faces and is particularly sensitive to negative emotional faces (29, 30). Individuals with SAD exhibit different characteristics when processing facial expressions compared to non-socially anxious people. Previous research findings indicated that individuals with high social anxiety exhibit shorter latency and greater amplitude of N170 in response to threat-related facial stimuli compared to those with low social anxiety (31). These results suggest that SAD individuals show significant early sensitivity to facial emotions. The impact of social anxiety on the interpretation of angry facial expressions, and whether therapeutic intervention can rectify these processing abnormalities, are still open questions.

The late positive potential (LPP), unlike N170, is a later component associated with emotional stimuli (32) and can be used as an indicator of top-down mechanisms when facing emotional stimuli (33). The LPP is thought to reflect continuous attention to emotional stimuli (34). Comparing positive and negative pictures with neutral pictures, an increased LPP amplitude was found (30), indicating that the recognition of emotional pictures is often accompanied by a greater allocation of attentional resources. The LPP amplitude of threat expressions was greater than that of affirmative expressions in the high-anxiety group, but not in the low-anxiety group, suggesting that the high-anxiety group processed negative expressions at a deeper perceptual level (35). Some studies have also shown that aversive expressions elicit greater LPP amplitude in people with high social anxiety (36, 37). Consequently, the LPP emerges as a dependable indicator of emotional reactivity in individuals with SAD. In conclusion, the N170 and LPP could be used to assess changes in social anxiety before and after treatment.

In the treatment of SAD, attentional bias is an important target. We administered hypnotic suggestions that targeted both the social anxiety symptoms and attention bias of individuals with SAD. The odd-one-out visual search task was used to assess attentional bias, including facilitated processing of negative emotional stimuli and difficulty disengaging attention from negative emotional stimuli (38). This paradigm requires participants to make judgments from a matrix of stimuli that are either the same or different. Anxious individuals exhibit rapid recognition of threatening stimuli and slowed disengagement (38).

In the present study, we aimed to evaluate the effects of 6 weeks of hypnotherapy on social anxiety, attention bias (measured using the odd-one-out visual search task), and EEG recordings measuring participants’ attentional bias and processing of different emotional faces. We hypothesized that hypnotherapy would alleviate social anxiety and decrease attention bias, as reflected by the odd-one-out task. We also expected to observe improvements in attention bias on the odd-one-out task using ERPs. We hypothesized that hypnosis might impact both early and late ERP amplitudes (N170 and LPP) in the odd-one-out visual search tasks. Furthermore, we predicted that reductions in social anxiety would be closely correlated with changes in both early and late components, as indexed by N170 and LPP.

## 2 Methods

### 2.1 Participants

Based on their social anxiety ratings on the Chinese version of the Liebowitz Social Anxiety Scale (LSAS), a sample of 638 undergraduate students was selected for screening (39). Those participants scoring above 63 were randomly assigned to the experimental group (11 males and 12 females, age: *M* = 19.57 years, *SD* = 0.72 years) or the control group (14 males and 9 females, age: *M* = 19.48 years, *SD* = 0.90 years), while those scoring less than 16 points were included in the baseline group (8 males and 15 females, age: *M* = 19.57 years, *SD* = 0.51 years). We set the cut-off point at 63 instead of 30 because it does not produce false positive identifications in non-SAD individuals (40). It was not practicable to blind therapists or participants to hypnosis and control allocations due to the nature of the intervention. The experimental group scored 7.29 ± 1.29 on the SHSS, the control group scored 7.62 ± 1.42, and the baseline group scored 7.38 ± 1.40. A one-way ANOVA was conducted on the hypnotic susceptibility scores of the three groups, revealing no significant difference between them (*P* = 0.532). Participants had normal or corrected-to-normal vision. Exclusion criteria included the presence of neurological illness, nervous system diseases, depression, severe medical diseases, color blindness, or color weakness. The study was approved by Human Ethics Committee of Anhui Medical University, with a protocol number of 2019H011. This study utilized a randomized controlled trial design, with the study identified by the code ChiCTR1900022651. Informed consent was obtained from all participants, and their participation was rewarded with 100 Yuan.

### 2.2 Procedure

**Figure 1:**
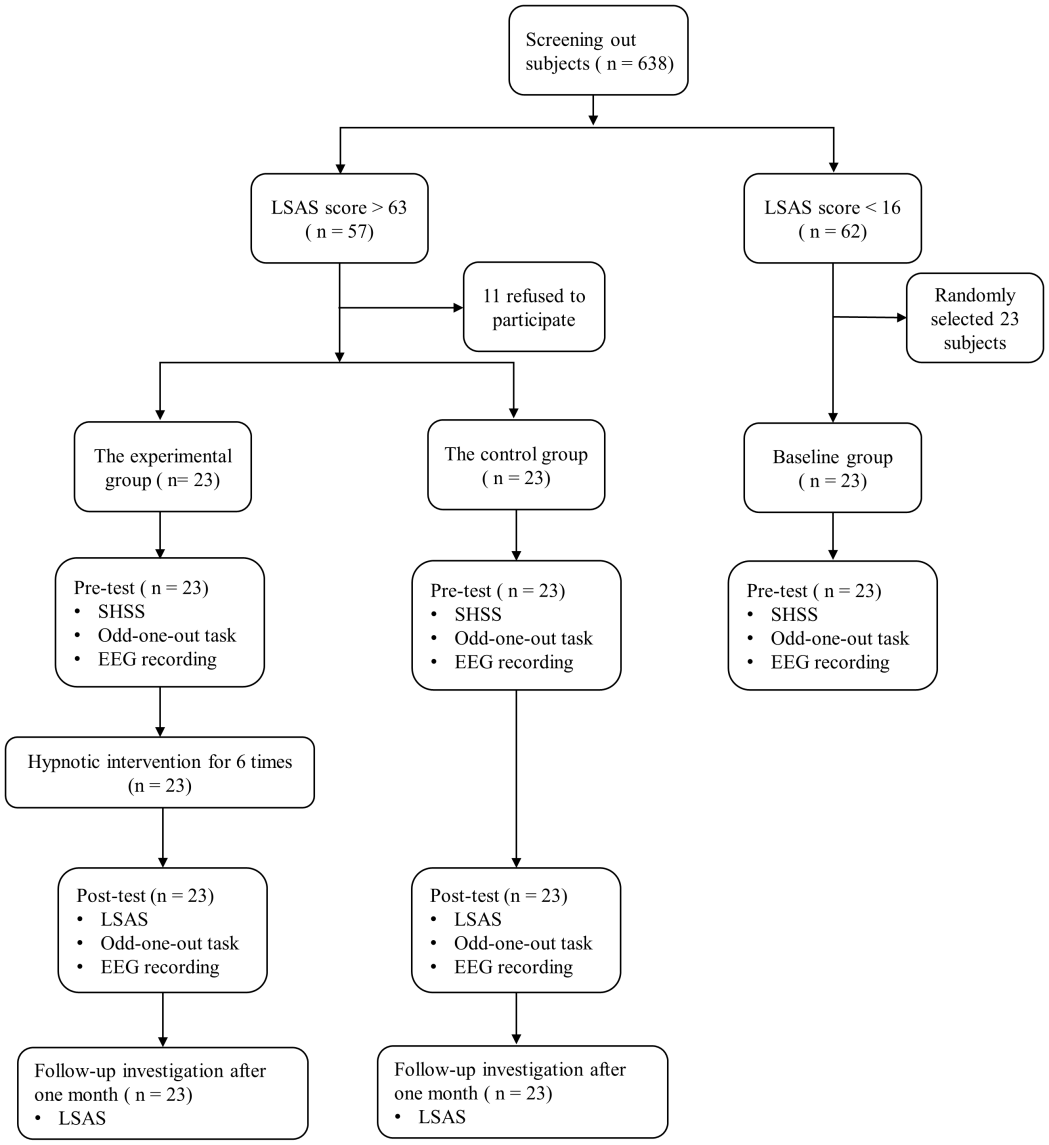
Flow chart of the enrollment and study.

At the pretest, participants completed two scales: the LSAS for screening purposes, and the Stanford Hypnotic Susceptibility Scale (SHSS) (41). All participants then performed an odd-one-out task under EEG recording. This allowed the collection of ERP data. After one or two days of pretest assessments, participants in the experimental group received hypnosis intervention. The hypnotic intervention was administered once weekly over a span of 6 weeks, with each session lasting 30 minutes. There was no intervention given to the control group or baseline group. Approximately one or two days following the final session, participants from both the experimental and control groups completed a posttest that closely resembled those employed for the pretest. One month after the posttest, both the experimental and control groups completed the LSAS. In this study, both the control and experimental groups participated in sessions of identical duration and with similar levels of researcher engagement. The protocol was standardized to ensure that all participants received consistent attention and had equivalent opportunities for interaction. **Figure 1** summarizes the experimental flow.

### 2.3 Stimuli and odd-one-out experimental paradigm

The face stimuli consisted of black lines on a white background, depicting angry, happy, and neutral expressions. The facial features, including the nose and outlines of the face, were constructed using one-pixel lines. The eyebrows, eyes, and mouth were rendered using two-pixel lines. This type of face stimulus can reduce the influence of specific temperament and instance effects associated with real faces (42).

To assess the attentional sensitivity and attention disengagement slowing for anxious individuals in response to threatening stimuli, an odd-one-out task was used. Attentional sensitivity to different face stimuli was measured by reaction time when selecting one angry or happy face against a background of five neutral faces, while difficulty of attention disengagement to different face stimuli was measured by reaction time when selecting one neutral face against a background of five angry or happy faces.

**Figure 2:**
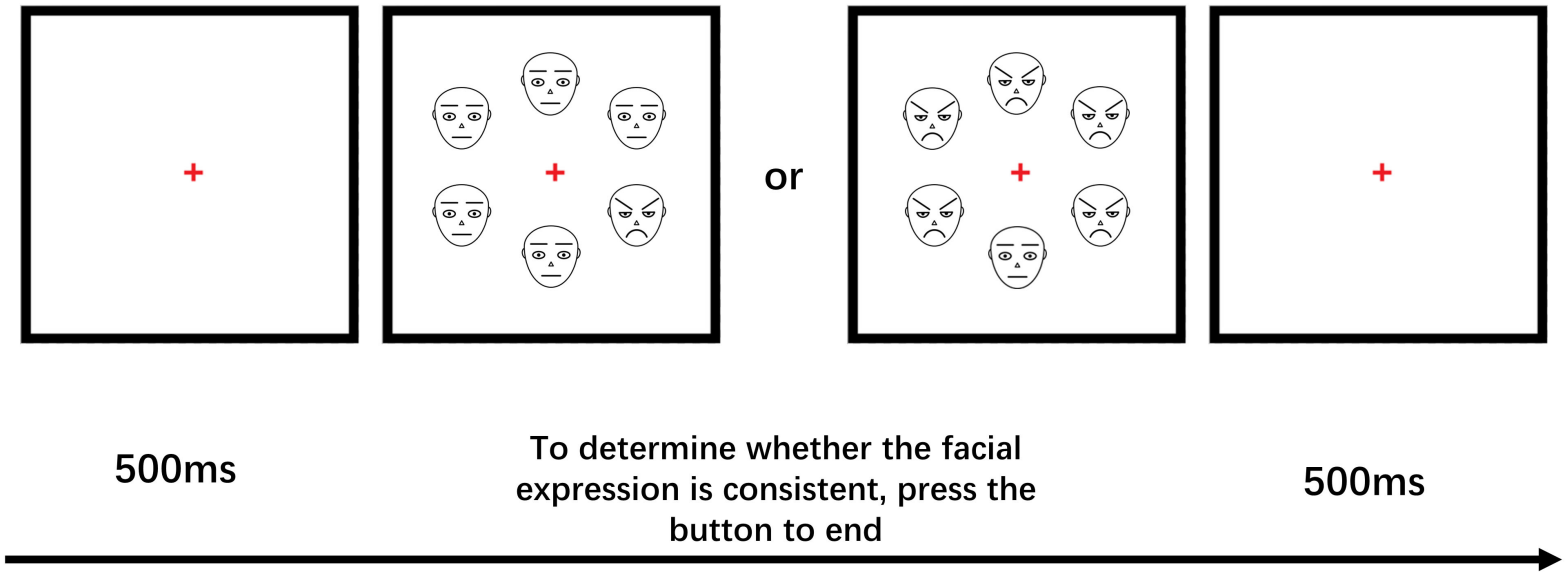
Schematic examples of trials used in each block.

The experimental stimuli were presented and behavioral data was recorded using the E-prime 2.0 software. Participants viewed a series of face stimuli arranged in a circular pattern. Fixation points were presented randomly for 500ms on the computer screen, followed by pictures of different faces (1 emotional face and 5 neutral faces, or 6 neutral faces; 1 neutral face and 5 emotional faces, or 6 emotional faces) presented for 2000ms-3000ms. Participants were instructed to judge whether the pictures of 6 faces were all the same and press the “A” key if they were the same, or the “L” key if they were different (see **Figure 2**). Emotional faces were randomly presented in one of six locations against a neutral face background during the attention sensitivity task, and neutral faces appeared in one of six locations against an emotional face background during the attention disengagement task. Prior to the formal experiment, a 36-trial practice experiment was conducted. The formal experiment consisted of two tasks, one testing attention sensitivity and the other measuring the difficulty of attention disengagement, each with 360 trials. Participants were counterbalanced between the tasks and took a 3-minute break between them.

### 2.4 Hypnotic intervention

The experimental group received six 30-minute hypnotic interventions, once a week, for one and a half months. The experimental environment was maintained in a quiet and clean state, with an experienced hypnotist guiding the participants to adopt the most relaxed sitting position possible in a chair. Then the hypnotist engages in a conversation with the participant to alleviate any concerns and establish a collaborative relationship. For example, they might say: “Feeling nervous during your first hypnosis session is common because it’s unknown territory, and it’s only natural to be curious about hypnosis. We hope you will actively participate.” or “We will not ask you to do anything that would make you look foolish or silly. Our purpose here is a serious scientific endeavor.” During the hypnotic induction (43), the hypnotherapist sits behind the participant on their left side, guiding them to gradually relax and close their eyes through soft verbal cues, leading to the hypnotic state. After the induction, the hypnotherapist presents standardized hypnotic suggestions verbally (see **Supplementary Material**). The subjects were gradually exposed to social anxiety from mild to severe, while being suggested to feel relaxed and happy at the same time. Hypnotic suggestions were used to change their attention and cognition toward the social anxiety situation while being told to feel relaxed and happy when exposed to social anxiety. The control and baseline groups did not receive any intervention.

### 2.5 EEG recording, pre-processing, and analysis

A 64-channel NeuroScan system (NeuroScan Inc., Herndon, VA) was used to record EEG data. The right and left mastoids were used as reference point. The vertical EOG was recorded above and below the left eye socket, while the horizontal EOG was recorded at the edges of the left and right eye sockets. The ERP recording started after the impedance of all channels was kept below 5 kΩ. EEG samples were bandpass filtered using a sampling rate of 0.1-30 Hz and continuously sampled at 500 Hz per channel. EEG was epoched offline into 1000 ms intervals, from 100 ms before to 900 ms after stimulus onset. The Neuroscan software was employed to develop a regression procedure for the offline removal of eye movement artifacts, specifically blinking and eye movement. Average amplitude of face types (angry vs. happy) and target stimuli (attention sensitivity vs. attention disengagement) were calculated, respectively.

Based on prior work, the N170 was evaluated at P7 and P8, where it was the most prominent (44), as the average activity between 130 and 190 ms following stimulus onset. The LPP was measured as the average activity from 400 to 600 ms after the stimulus at P1, Pz, and P2 (45, 46).

During the test, participants are required to complete a total of 360 trials: 180 under each condition of attentional sensitivity and attentional disengagement, with each condition further divided into 60 angry faces (sensitive to angry faces/disengaging from angry faces), 60 happy faces (sensitive to happy faces/disengaging from happy faces), and 60 congruent faces (all neutral faces/all emotional faces). The mean number of valid epochs for the experimental group was 44.54 for attention sensitivity on angry faces, 43.28 for attention sensitivity on happy faces, 41.75 for attention disengagement on angry faces and 40.35 for attention disengagement on happy faces and 38.85 for congruent faces. The mean number of valid epochs for the control group was 44.54 for attention sensitivity on angry faces, 39.26 for attention sensitivity on happy faces, 40.12 for attention disengagement on angry faces and 40.56 for attention disengagement on happy faces and 38.72 for congruent faces. All data were analyzed using SPSS 18.0. The significance level was set at *p* < 0.05. To compare the experimental and control groups with the baseline group on N170 and LPP amplitude, repeated-measures ANOVAs were performed with the between-group factor of Group (experimental/control/baseline) and two within-group factors: face (anger/happiness) and electrode (P7/P8, P1/Pz/P2). In the second set of analyses, a within-group therapy factor (pre/post) was introduced to evaluate the effects of the therapy. Paired and independent samples t-tests were used to further assess significant interactions in each component. To investigate relationship between ERP changes and symptom improvement, we then run correlation analysis. The study selected demographic variables including age, gender, and SHSS scores as covariates, which are considered important confounding factors that could influence the research outcomes. Bonferroni correction was employed to control for multiple comparison problem. A Greenhouse-Geisser correction was applied to adjust the degrees of freedom and F-value for the ANOVA.

## 3 Results

### 3.1 Behavioral performance

To test whether our hypnotherapy improved social anxiety symptoms, We conducted a 2 (group: experimental and control) × 3 (time: pretest, posttest, and follow-up) repeated measures ANOVA to examine the differences in LSAS scores between the two groups (group: *F*_(1, 44)_ = 126.26, *P* < 0.01; time: *F*_(2, 43)_ = 96.90, *P* < 0.01). The group × time interaction was significant (*F*_(2, 43)_ = 81.21, *P* < 0.01). Specifically, the experimental group tended to have lower score on the LSAS after hypnosis treatments (*t*_(22)_ = 11.98, *P* < 0.01). Moreover, the difference in LSAS scores between posttest and follow-up was significant (*t*_(22)_ = 4.47, *P* < 0.01), suggested that the effect of the hypnotic intervention continued and that there was a long-term effect.

Under the attention sensitivity conditions, a 2 (time: pretest, posttest) × 2 (face: angry, happy) × 2 (group: experimental group, control group) mixed-model ANOVA was conducted. The ANOVA on RTs revealed main effects of time (*F*_(1,44)_ = 26.66, *P* < 0.01), group (*F*_(1,44)_ = 21.38, *P* < 0.01) and face (*F*_(1,44)_ = 152.87, *P* < 0.01). There was a significant interaction between time and group (*F*_(1,44)_ = 32.32, *P* < 0.01). Simple effect analysis showed that the experimental group’s RTs to angry and happy faces were significantly lower in the pretest than in the posttest (angry: *t*_(22)_ = -7.00, *P* < 0.01; happy: *t*_(22)_ = -6.12, *P* < 0.01). The control group showed no significant difference in RTs between the pretest and posttest (angry: *t*_(22)_ = 0.33, *P* = 0.745; happy: *t*_(22)_ = 0.48, *P* = 0.633) (**Table 1**).

**Table 1:** Mean reaction times and accuracy rates as a function of attention sensitivity condition ( $\bar{\text{x}}$ ± SD).

Emotional faces	Experimental Group		Control Group	
	Pretest	Posttest	Pretest	Posttest
	Reaction time (ms)			
Angry	612.00 ± 49.83	767.45 ± 87.83	619.63 ± 61.46	613.97 ± 69.14
Happy	663.53 ± 68.12	810.38 ± 97.14	675.75 ± 63.39	666.87 ± 70.13
	Accuracy rate (%)			
Angry	82.75 ± 7.46	86.09 ± 5.07	82.46 ± 6.61	81.45 ± 6.87
Happy	81.88 ± 7.91	82.17 ± 6.57	81.59 ± 5.46	81.74 ± 8.36

Under the attention disengagement conditions, a 2 (time: pretest, posttest) × 2 (face: angry, happy) × 2 (group: experimental group, control group) mixed-model ANOVA was conducted. The ANOVA on RTs revealed main effects of time (*F*_(1,44)_ = 33.98, *P* < 0.01), group (*F*_(1,44)_ = 10.65, *P* < 0.01) and face (*F*_(1,44)_ = 10.10, *P* < 0.01). There were significant interactions between time and group (*F*_(1,44)_ = 51.52, *P* < 0.01),face and group (*F*_(1,44)_ = 4.92, *P* = 0.03),time and face (*F*_(1,44)_ = 5.30, *P* = 0.026). Simple effect analysis showed that the experimental group’s RTs to angry and happy faces were significantly higher in the pretest than in the posttest (angry: *t*_(22)_ = 7.80, *P* < 0.01; happy: *t*_(22)_ = 6.57, *P* < 0.01). The control group showed no significant difference in RTs between the pretest and posttest (angry: *t*_(22)_ = -0.99, *P* = 0.335; happy: *t*_(22)_ = -1.58, *P* = 0.129). The aforementioned results imply that, after hypnotherapy, the experimental group’s attentional sensitivity to emotional faces decreased, and the difficulty in disengaging attention reduced (**Table 2**).

**Table 2:** Mean reaction times and accuracy rates as a function of attention disengagement condition ( $\bar{\text{x}}$ ± SD).

Emotional faces	Experimental Group		Control Group	
	Pretest	Posttest	Pretest	Posttest
	Reaction time (ms)			
Angry	1307.99 ± 145.23	1023.77 ± 154.72	1246.97 ± 104.08	1266.18 ± 100.48
Happy	1260.43 ± 154.72	1016.19 ± 147.68	1233.88 ± 120.32	1269.46 ± 91.21
	Accuracy rate (%)			
Angry	81.30 ± 4.79	82.46 ± 4.53	82.32 ± 5.24	83.19 ± 5.34
Happy	83.33 ± 4.91	82.75 ± 6.34	82.61 ± 7.09	82.90 ± 5.14

### 3.2 ERP results

#### 3.2.1 Pretest N170

Under the attention sensitivity conditions, a 2 (electrode: P7, P8) × 2 (face: angry vs. happy) × 3 (group: the experimental group, the control group, baseline group) mixed-model ANOVA was conducted. The analysis revealed a main effect of group (*F*_(2,66)_ = 4.25, *P* = 0.018, *η^2^_p_* = 0.114), electrode (*F*_(1,66)_ = 13.88, *P* < 0.001, *η^2^_p_* = 0.174) and face (*F*_(2,66)_ = 7.58, *P* = 0.001, *η^2^_p_* = 0.187). There was a significant interaction between face and electrode (*F*_(1,66)_ = 9.17, *P* = 0.003, *η^2^_p_* = 0.122). Simple effect analysis showed that there were significant differences among the three groups in their responses to angry faces at electrode at P7 and P8 (P7: *F*_(2,66)_ = 43.79, *P* < 0.001, *η^2^_p_* = 0.570; P8: *F*_(2,66)_ = 36.67, *P* < 0.001, *η^2^_p_* = 0.526). From the P7 electrode, the experimental group exhibited higher N170 amplitude compared to the control (*P* < 0.05) and baseline groups (*P* < 0.05), whereas the control and baseline groups showed no significant difference(*P* = 0.07); from the P8 electrode, there was no significant difference in N170 amplitude between the experimental and control groups (*P* = 0.134), both of which were significantly higher than the baseline group (*P* < 0.05). The three groups also exhibited significant differences in their neural responses to happy faces at P7 and P8 (P7: *F*_(2,66)_= 42.14, *P* < 0.001, *η^2^_p_* = 0.516; P8: *F*_(2,66)_ = 41.23, *P* < 0.001, *η^2^_p_* = 0.556). From the P7 electrode, there was no significant difference in N170 amplitude between the experimental and control groups (*P* = 0.143), both of which were significantly higher than the baseline group (*P* < 0.05).

Under the attention disengagement conditions, a 2 (electrode: P7, P8) × 2 (face: angry vs. happy) × 3 (group: the experimental group, the control group, baseline group) mixed-model ANOVA was conducted. The analysis revealed a main effect of group (*F*_(2,66)_ = 107.89, *P* < 0.001, *η^2^_p_* = 0.766). For angry faces, there was no significant difference in N170 amplitude between the experimental and control groups, and both were higher than the baseline group (*P* < 0.05). For happy faces, at the P7 electrode, there was no significant difference in N170 amplitude between the experimental and control groups (*P* = 0.203), and both were higher than the baseline group (*P* < 0.05); at the P8 electrode site, the experimental group’s N170 amplitude was not significantly different from that of the lower group (*P* = 0.132), and both were higher than the control group (*P* < 0.05).

#### 3.2.2 Pretest LPP

Under the attention sensitivity conditions, a 3 (electrode: P1, Pz, P2) × 2 (face: angry vs. happy) × 3 (group: the experimental group, the control group, baseline group) ANOVA was conducted. The analysis revealed a main effect of group (*F*_(2,66)_ = 83.552, *P <* 0.001, *η^2^_p_* = 0.717), electrode (*F*_(2,132)_ = 11.483, *P <*0.001, *η^2^_p_* = 0.148) and face (*F*_(1,66)_ = 16.229, *P <* 0.001, *η^2^_p_* = 0.197). There was a significant interaction between face and electrode (*F*_(2,132)_ = 5.853, *P =* 0.004, *η^2^_p_* = 0.081). Simple effects analysis revealed that there were statistically significant differences among the three groups in their neural responses to angry faces at different electrode sites (*F*_(2,136)_ = 6.519, *P* = 0.002, *η^2^_p_* = 0.087). For angry faces, there was no significant difference in LPP amplitude between the experimental and control groups (P1: *P* = 0.102; P2: *P* = 0.009; Pz: *P* = 0.491), and both were higher than the baseline group (*P* < 0.001). Similarly, the groups showed statistically significant differences in response to happy faces across different electrode sites (*F*_(2,136)_ = 9.579, *P <* 0.001, *η^2^_p_* = 0.123). There was no significant difference in LPP amplitude between the experimental and control groups (P1: *P* = 0.269; P2: *P* = 0.114;Pz: *P* = 0.561), and both were higher than the baseline group (*P* < 0.001).

Under the attention disengagement conditions, a 3 (electrode: P1, Pz, P2) × 2 (face: angry vs. happy) × 3 (group: the experimental group, the control group, baseline group) ANOVA was conducted. The analysis revealed a main effect of group (*F*_(1,66)_ = 73.200, *P <* 0.001, *η^2^_p_* = 0.689) and face (*F*_(1,66)_ = 12.137, *P =* 0.01, *η^2^_p_* = 0.155). Additionally there was a significant interaction between electrode, face and group (*F*_(4,132)_ = 2.582, *P <* 0.040, *η^2^_p_* = 0.073). There was a significant interaction between face and group (*F*_(2,66)_ = 4.314, *P =* 0.017, *η^2^_p_* = 0.116). Simple effects analysis conducted on the face variable indicated that there were statistically significant differences among the three groups in their neural responses to angry faces at electrode sites P1, PZ, and P2 (P1: *F*_(2,68)_ = 52.427, *P <* 0.001, *η^2^_p_* = 0.606; P2: *F*_(2,68)_ = 44.265, *P <* 0.001, *η^2^_p_* = 0.573; P3: *F*_(2,68)_ = 34.192, *P <* 0.001, *η^2^_p_* = 0.509). For angry faces, there was no significant difference in LPP amplitude between the experimental and control groups (P1: *P* = 0.267; P2: *P* = 0.465; Pz: *P* = 0.234), and both were higher than the baseline group (*P* < 0.001). In response to happy faces, there was no significant difference between the experimental and control groups (P1: P = 0.078; P2: P = 0.269; Pz: P = 0.916), and both were higher than the baseline group (P < 0.001).

#### 3.2.3 Posttest N170

**Figure 3:**
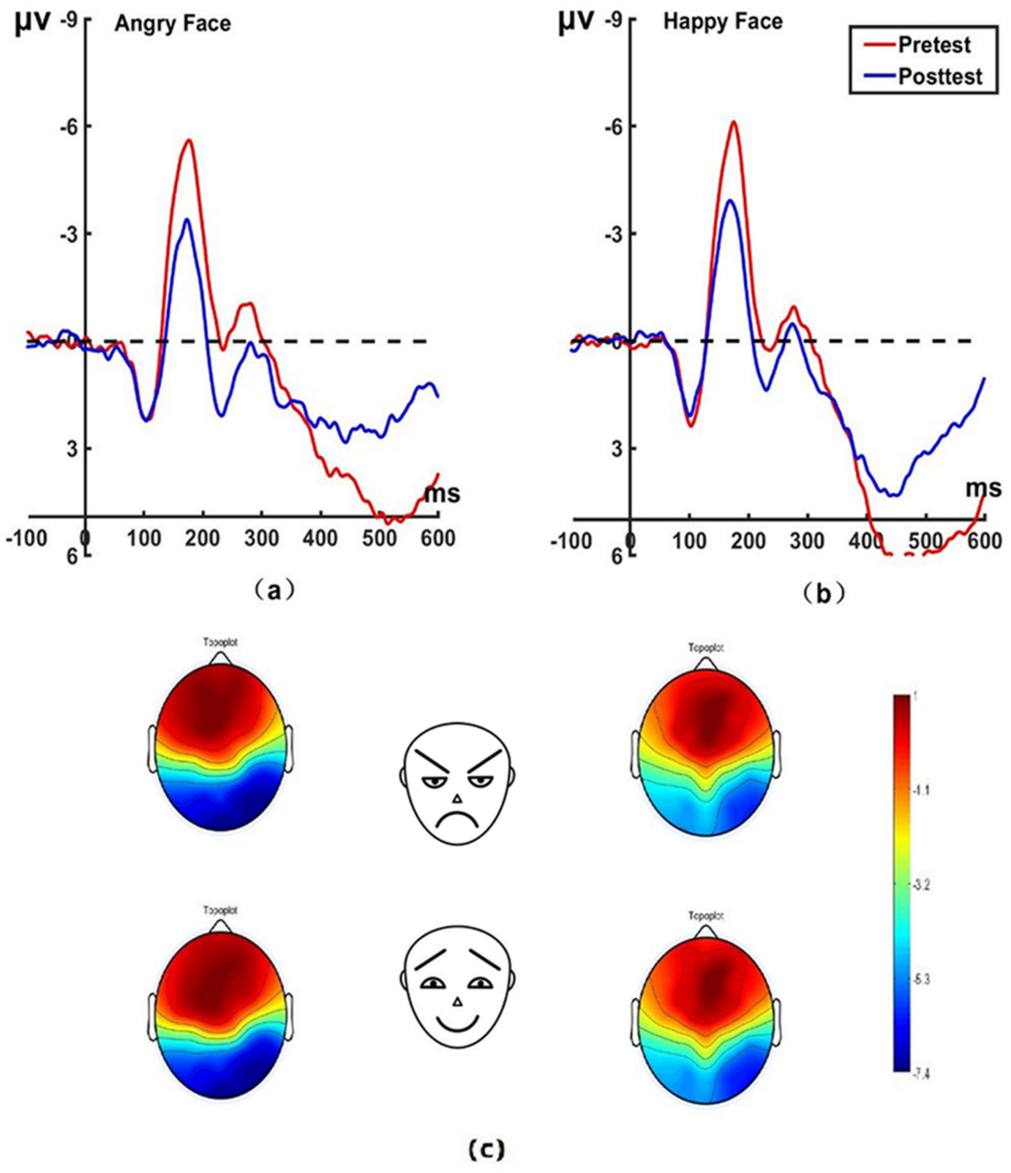
Pretest and posttest N170 analyses were conducted on the P7 electrode for the experimental group. **(A)** Grand average waveforms of the N170 for angry faces under the attention sensitivity conditions; **(B)** Grand average waveforms of the N170 for happy faces under the attention sensitivity conditions; **(C)** Scalp topographies of the N170 component over different faces and times. The left side shows data collected before the hypnotherapy, and the right side shows data collected after the hypnotherapy. ms, Millisecond.

**Figure 4:**
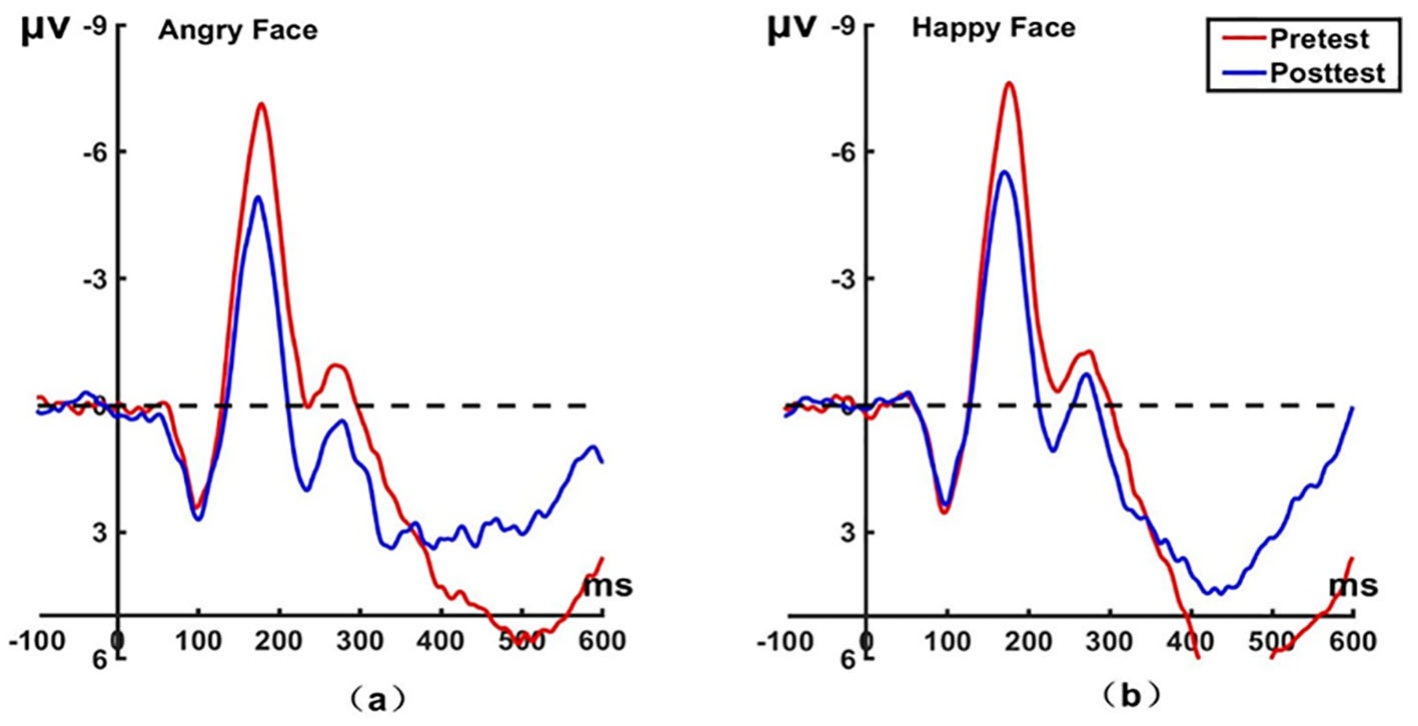
Pretest and posttest N170 analyses of the P8 electrode for the experimental group. **(A)** Grand averages waveforms of the N170 for angry faces under the attention sensitivity conditions. **(B)** Grand averages waveforms of the N170 for happy faces under the attention sensitivity conditions. ms, Millisecond.

Under the attention sensitivity conditions, we conducted a mixed-model ANOVA with factors of electrode (P7, P8), face (angry vs. happy), group (experimental group, control group), and time (pretest, posttest). There were significant main effect of time (*F*_(1,44)_ = 6.89, *P* = 0.012, *η^2^_p_* = 0.135), group (*F*_(1,44)_ = 7.06, *P* = 0.010, *η^2^_p_* = 0.138) and face (*F*_(1,44)_ = 17.45, *P* < 0.001, *η^2^_p_* = 0.010). A significant Group × Time interaction was found for the N170 (*F*_(1,44)_ = 8.32, *P* = 0.006, *η^2^_p_* = 0.159). Simple effect analysis showed a marked decrease in N170 amplitudes for both angry (P7: *t*_(22)_ = 5.53, *P* < 0.001, Cohen’s *d* = 0.763; P8: *t*_(22)_ = 4.06, *P* < 0.001, Cohen’s *d* = 0.654) and happy faces (P7: *t*_(22)_ = −3.12, *P* = 0.005, Cohen’s *d* = 0.553; P8: *t*_(22)_ = 2.11, P = 0.046, Cohen’s *d* = 0.410) for the experimental group. While there was no significant change in N170 amplitude for both angry (P7: *t*_(22)_ = 0.12, *P* = 0.905, Cohen’s *d* = 0.025; P8: *t*_(22)_ = 0.21, *P* = 0.835, Cohen’s *d* = 0.044) and happy faces (P7: *t*_(22)_ = 0.76, *P* = 0.455, Cohen’s *d* = 0.159; P8: *t*_(22)_ = 0.62, *P* = 0.542, Cohen’s *d* = 0.131) for the control group on pretest and posttest. No significant differences were found between the experimental and control group on the N170 amplitudes before treatment. The brain topographic map, as shown in **Figure 3** (P7 electrode; P8 electrode see **Figure 4**), indicate that when the N170 component was evoked by stimulation, significant negative waves appeared in the right occipitotemporal lobe. However, the area was smaller and less excitatory than before hypnotherapy.

**Figure 5:**
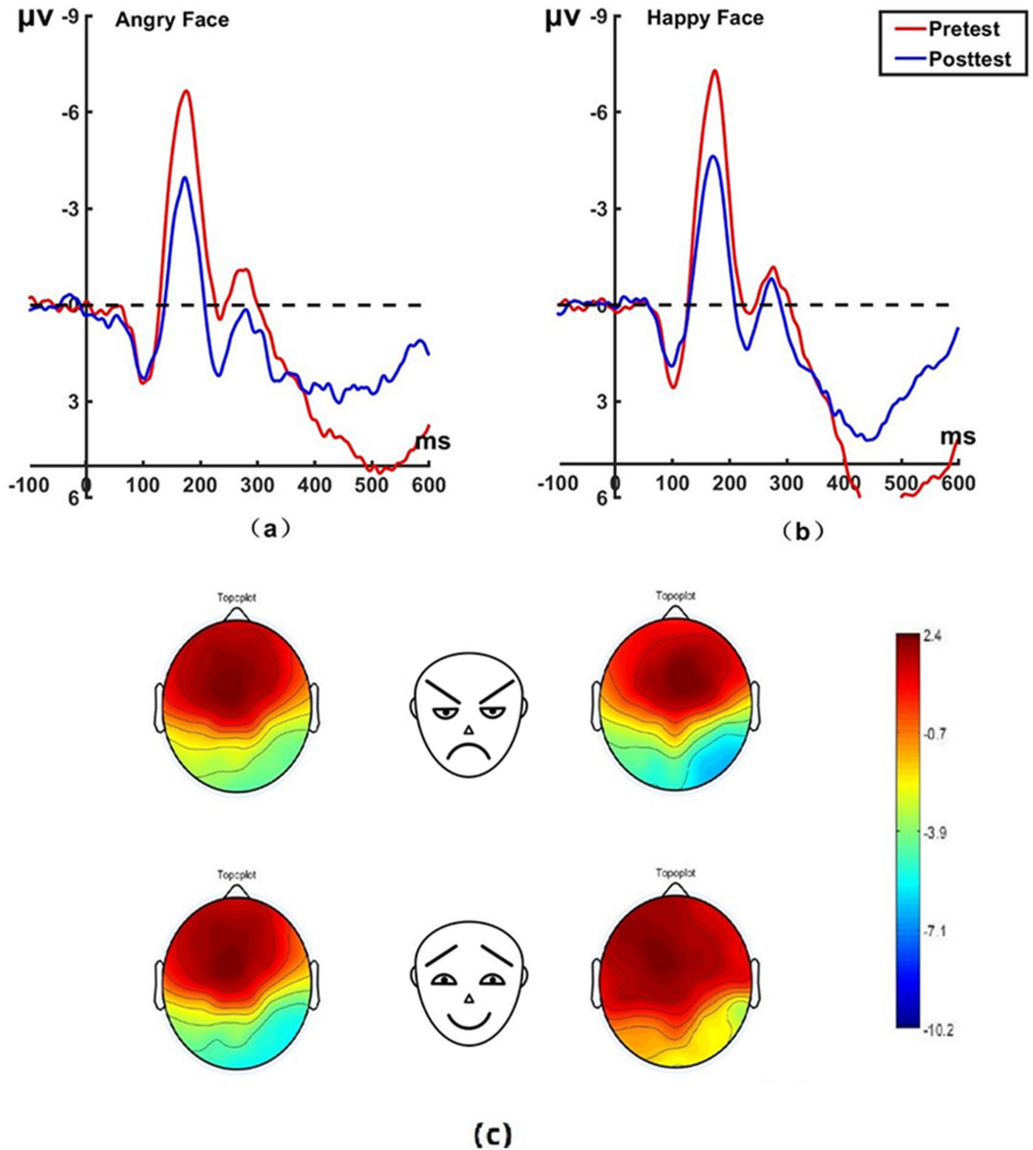
Pretest and posttest N170 analyses of the P7 electrode for the experimental group. **(A)** Grand average waveforms of the N170 for angry faces under the attention disengagement conditions; **(B)** Grand average waveforms of the N170 for happy faces under the attention disengagement conditions; **(C)** Scalp topographies of the N170 component over different faces and times. Left before the hypnotherapy. Right after the hypnotherapy. ms, Millisecond.

**Figure 6:**
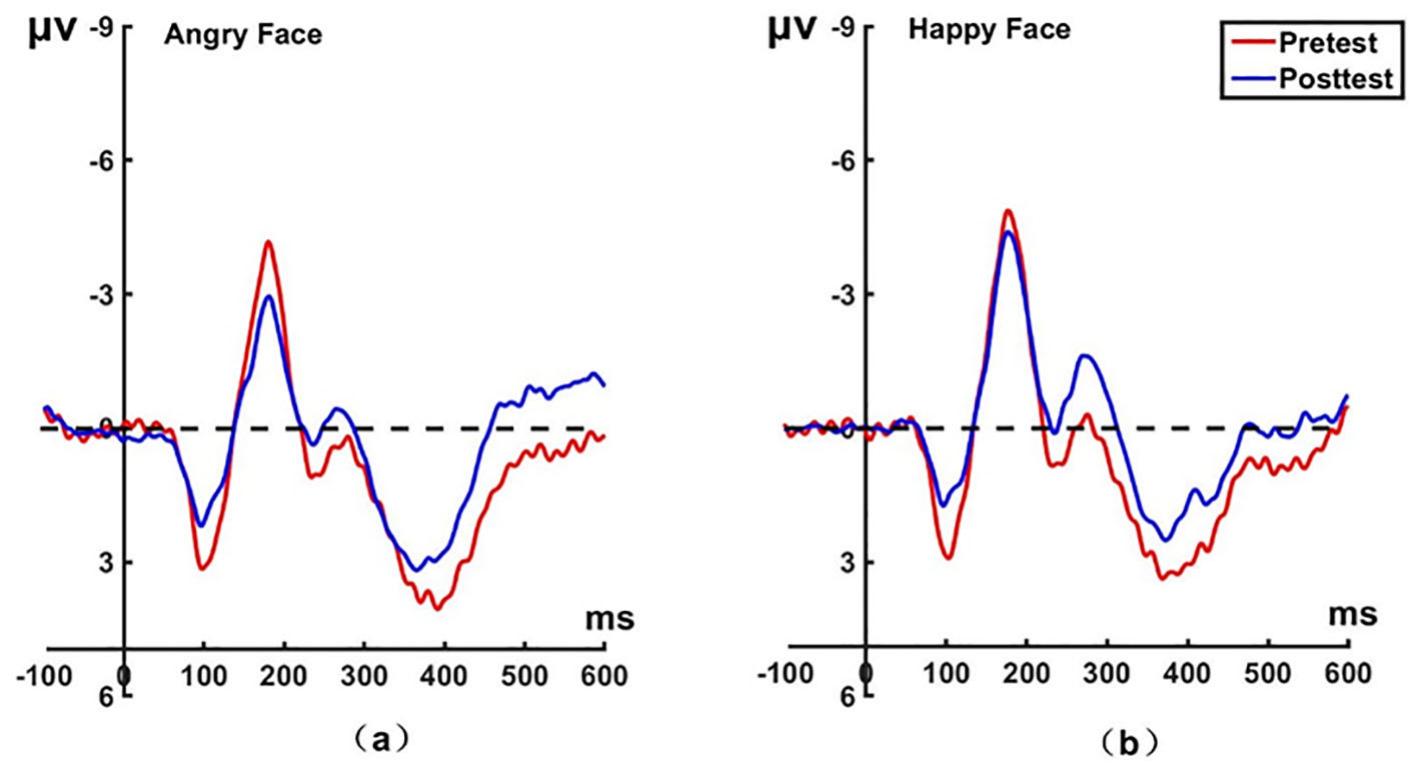
Pretest and posttest N170 analyses of the P8 electrode for the experimental group. **(A)** Grand averages waveforms of the N170 for angry faces under the attention disengagement conditions. **(B)** Grand averages waveforms of the N170 for happy faces under the attention disengagement conditions. ms, Millisecond.

Under the attention disengagement conditions, we conducted a mixed-model ANOVA with factors of electrode (P7, P8), face (angry vs. happy), group (experimental vs. control), and time (pretest vs. posttest). There were significant main effects of time (*F*_(1,44)_ = 8.832, *P* = 0.005, *η^2^_p_* = 0.167) and group (*F*_(1,44)_ = 3.891, *P* < 0.05, *η^2^_p_* = 0.081). A significant face × time interaction was found for the N170 (*F*_(1,44)_ = 6.67, *P* = 0.013, *η^2^_p_* = 0.132). Simple effect analysis showed that the experimental group had lower N170 amplitudes for angry faces after hypnosis (*t*_(22)_ = −2.972, *P* = 0.007, Cohen’s *d* = 0.535), while this pattern was not found for happy faces (*t_(22)_* = −0.008, *P* = 0.994, Cohen’s *d* = 0.001). For both angry and happy faces, no difference in N170 amplitude was found for pre and posttest in the control group at P7 (happy: *t*_(22)_ = 0.76, *P* = 0.455, Cohen’s *d* = 0.159; angry: *t*_(22)_ = 0.12, *P* = 0.905, Cohen’s *d* = 0.025) and P8 (happy: *t*_(22)_ = 0.62, *P* = 0.542, Cohen’s *d* = 0.131; angry: *t*_(22)_ = 0.21, *P* = 0.835, Cohen’s *d* = 0.044). In addition, at the P8 electrode, the experimental group differed in their N170 amplitude to angry and happy faces (*t*_(22)_ = 45.79, *P* < 0.001, Cohen’s *d* = 0.994) before hypnosis. The brain topographic map, as shown in **Figure 5** (P7 electrode; P8 electrode shown in **Figure 6**), indicates that significant negative waves appeared in the right occipitotemporal lobe when the N170 component was evoked by stimulation. However, the area was less activated than before hypnotherapy when disengaging attention from happy faces.

#### 3.2.4 Posttest LPP

Under the attention sensitivity conditions, a 3 (electrode: P1, Pz, P2) × 2 (face: angry vs. happy) × 2 (group: the experimental group, the control group) × 2 (time: pretest, posttest) mixed-model ANOVA was conducted. The analysis revealed a main effect of time (*F*_(1,44)_ = 21.874, *P <* 0.001, *η^2^_p_ =* 0.332) and electrode (*F*_(2,88)_ = 4.973, *P =* 0.009, *η^2^_p_ =* 0.102). There was a significant interaction between time, face, electrode, and group (*F*_(2,88)_ = 4.708, *P =* 0.011, *η^2^_p_=* 0.097).

**Figure 7:**
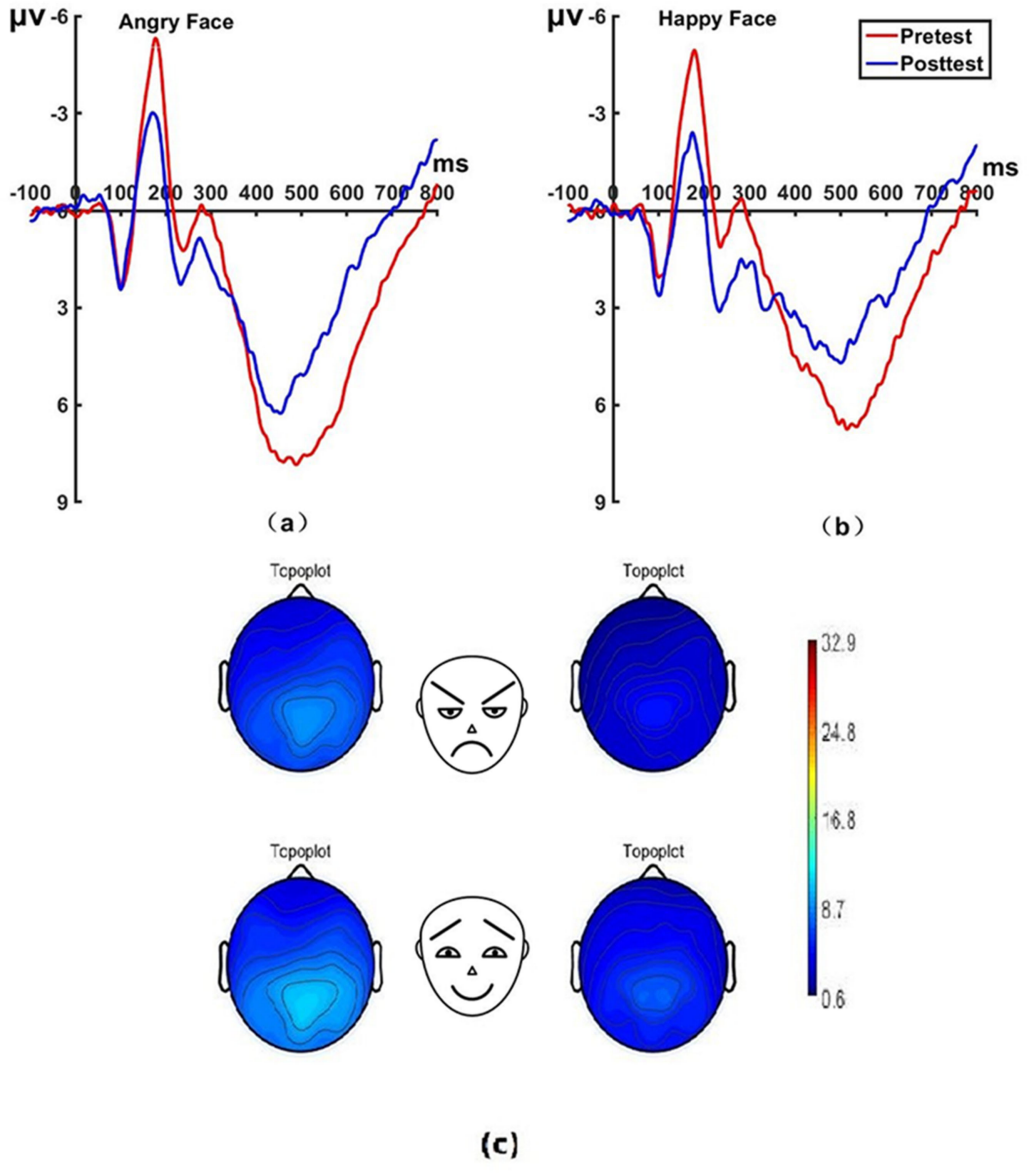
Pretest and posttest LPP analyses of the P1 electrode for the experimental group. **(A)** Grand average waveforms of the LPP for angry faces under the attention sensitivity conditions; **(B)** Grand average waveforms of the LPP for happy faces under the attention sensitivity conditions; **(C)** Scalp topographies of the LPP component over different faces and time. Left before the hypnotherapy. Right after the hypnotherapy. ms, Millisecond.

**Figure 8:**
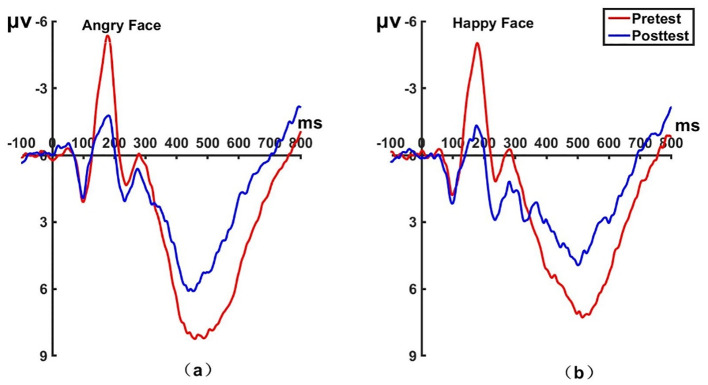
Pretest and posttest LPP analyses of the PZ electrode for the experimental group. **(A)** Grand averages waveforms of the LPP for angry faces under the attention sensitivity conditions. **(B)** Grand averages waveforms of the LPP for happy faces under the attention sensitivity conditions. ms, Millisecond.

**Figure 9:**
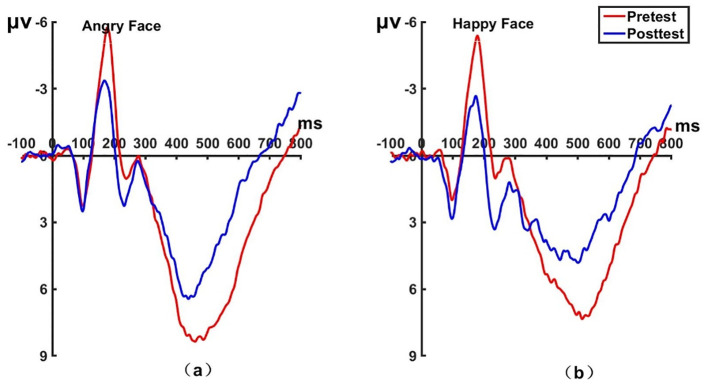
Pretest and posttest LPP analyses of the P2 electrode for the experimental group. **(A)** Grand averages waveforms of the LPP for angry faces under the attention sensitivity conditions. **(B)** Grand averages waveforms of the LPP for happy faces under the attention sensitivity conditions. ms, Millisecond.

There was a significant interaction between time, face and group (*F*_(1,44)_ = 8.194, *P =* 0.006, *η^2^_p_ =* 0.157). There was a significant interaction between time and face (*F*_(1,44)_ = 32.124, *P <* 0.001, *η^2^_p_ =* 0.422), and between face and group (*F*_(1,44)_ = 13.009, *P =* 0.001, *η^2^_p_ =* 0.228). Additionally, there was a marginal significant interaction between face, electrode and group (*F*_(2,88)_ = 2.960, *P =* 0.057, *η^2^_p_ =* 0.063). For the experimental group, the LPP of emotional facial stimuli was significantly lower at post-hypnosis than at pre-hypnosis (angry: *t*_(22)_ = 5.123, *P <* 0.001, Cohen’s *d* = 0.737; happy: *t*_(22)_ = 2.308, *P* = 0.031, Cohen’s *d* = 0.441). However, the LPP remained unchanged in the control group following hypnosis (angry: *t*_(22)_ = 1.116, *P* = 0.276, Cohen’s *d* = 0.231; happy: *t*_(22)_ = -1.175, *P* = 0.253, Cohen’s *d* = 0.243). The brain topographic map, as shown in **Figure 7** (P1 electrode; **Figure 8** for Pz electrode; **Figure 9** for P2 electrode), indicates that when the LPP component was evoked by stimulation, significant positive waves appeared in the right parietal lobe of the brain. After hypnotic intervention, the area was less excitable than before.

Under the attention disengagement conditions, a 3 (electrode: P1, Pz, P2) × 2 (face: angry vs. happy) × 2 (group: the experimental group, the control group) × 2 (time: pretest, posttest) mixed-model ANOVA was conducted. The analysis revealed main effect of time (*F*_(1,44)_ = 19.385, *P <* 0.001, *η^2^_p_ =* 0.306) and face (*F*_(1,44)_ = 5.373, *P* = 0.025, *η^2^_p_ =* 0.109).

**Figure 10:**
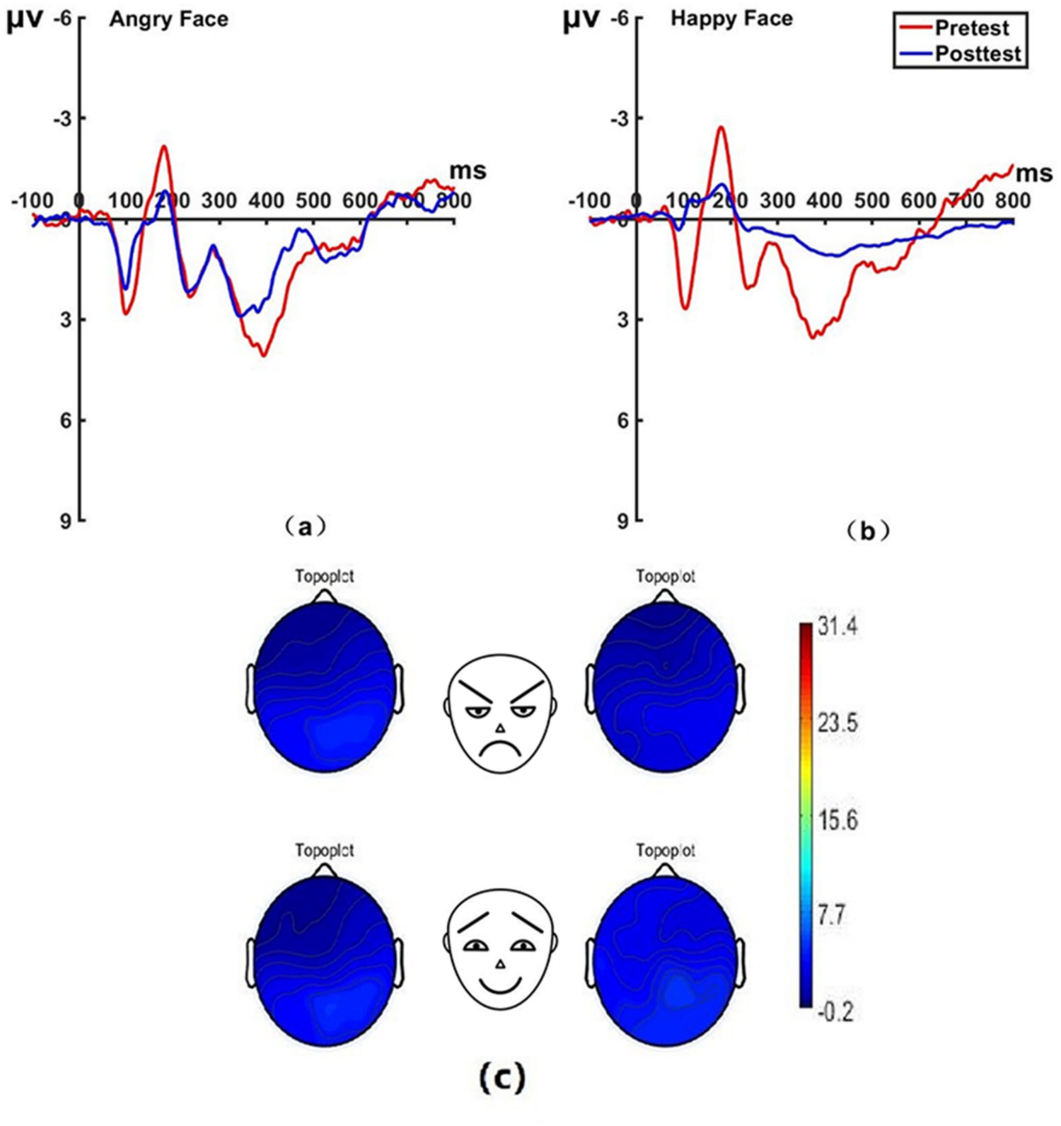
Pretest and posttest LPP analyses of the P1 electrode for the experimental group. **(A)** Grand averages waveforms of the LPP for angry faces under the attention disengagement conditions; **(B)** Grand averages waveforms of the LPP for happy faces under the attention disengagement conditions; **(C)** Scalp topographies of the LPP component over different faces and time. Left before the hypnotherapy. Right after the hypnotherapy. ms, Millisecond.

**Figure 11:**
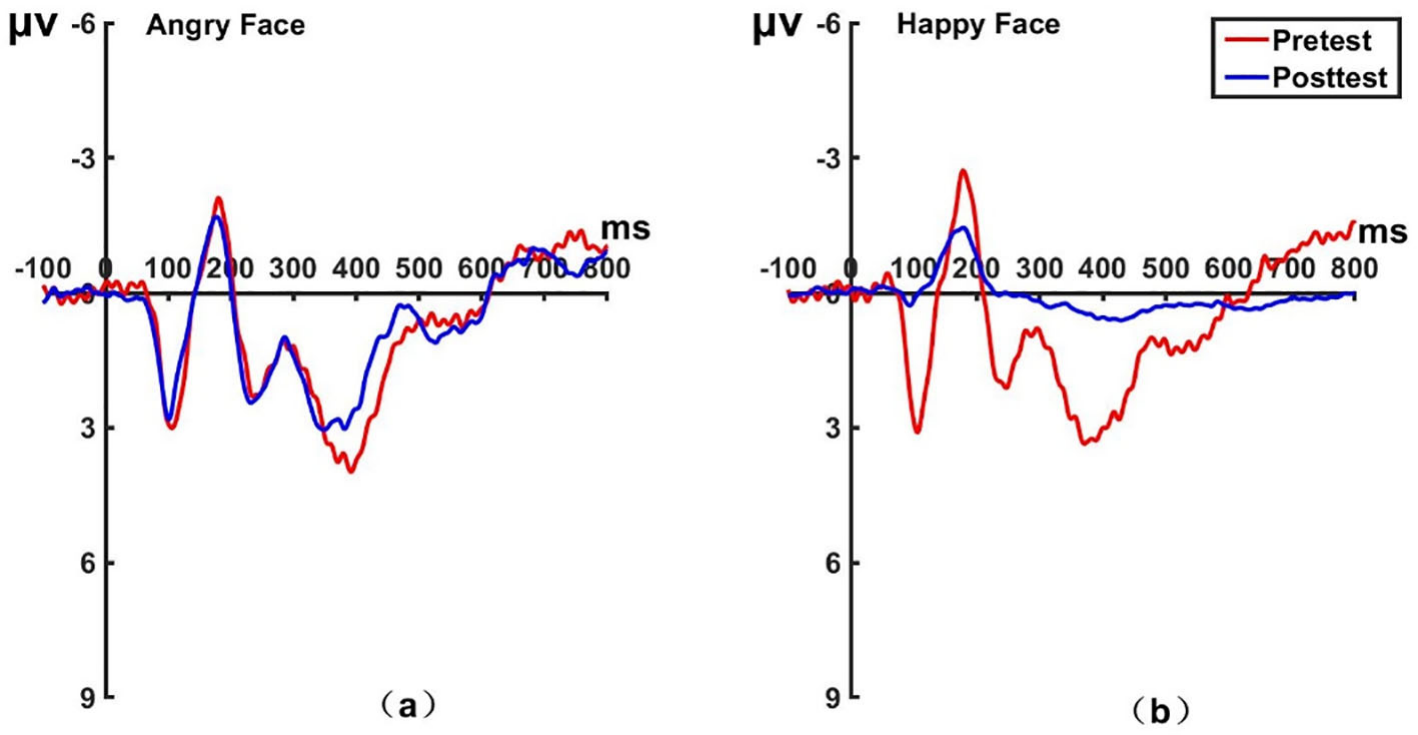
Pretest and posttest LPP analyses of the PZ electrode for the experimental group. **(A)** Grand averages waveforms of the LPP for angry faces under the attention disengagement conditions. **(B)** Grand averages waveforms of the LPP for happy faces under the attention disengagement conditions. ms, Millisecond.

**Figure 12:**
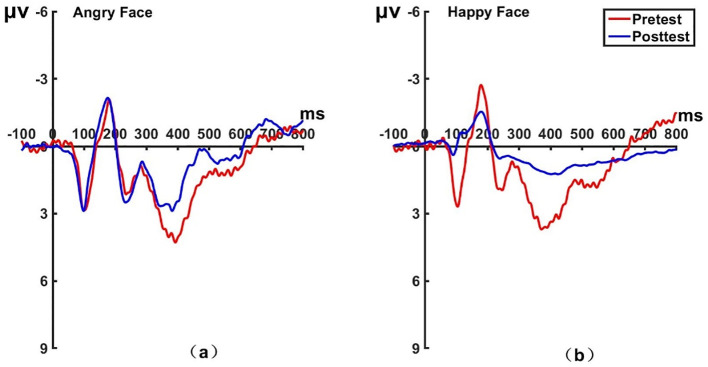
Pretest and posttest LPP analyses of the P2 electrode for the experimental group. **(A)** Grand averages waveforms of the LPP for angry faces under the attention disengagement conditions. **(B)** Grand averages waveforms of the LPP for happy faces under the attention disengagement conditions. ms, Millisecond.

A significant interaction of time, face, electrode, and group was found for the LPP amplitude (*F*_(2,88)_ = 3.120, *P =* 0.049, *η^2^_p_ =* 0.066). There was a significant interaction between time and face (*F*_(1,44)_ = 14.702, *P <* 0.001, *η^2^_p_ =* 0.250). For the experimental group, the LPP of angry faces at post-hypnosis was significantly lower than that at pre-hypnosis, while this pattern was not found for happy faces (angry: *t*_(22)_ = 3.719, *P* = 0.01, Cohen’s *d* = 0.621; happy: *t*_(22)_ = 1.232, *P* = 0.231, Cohen’s *d* = 0.254). For the control group, the LPP did not change after hypnosis (angry: *t*_(22)_ = 1.533, *P* > 0.05, Cohen’s *d* = 0.310; happy: *t*_(22)_ = -1.175, *P* = 0.253, Cohen’s *d* = 0.243). The brain topographic map is shown in **Figure 10** (P1 electrode; Pz electrode see **Figure 11**; P2 electrode see **Figure 12**). After hypnosis treatment, when subjects responded to happy faces, the left prefrontal cortex was more excitable than before.

### 3.3 Correlations between change in the LSAS scores and ERP results

**Figure 13:**
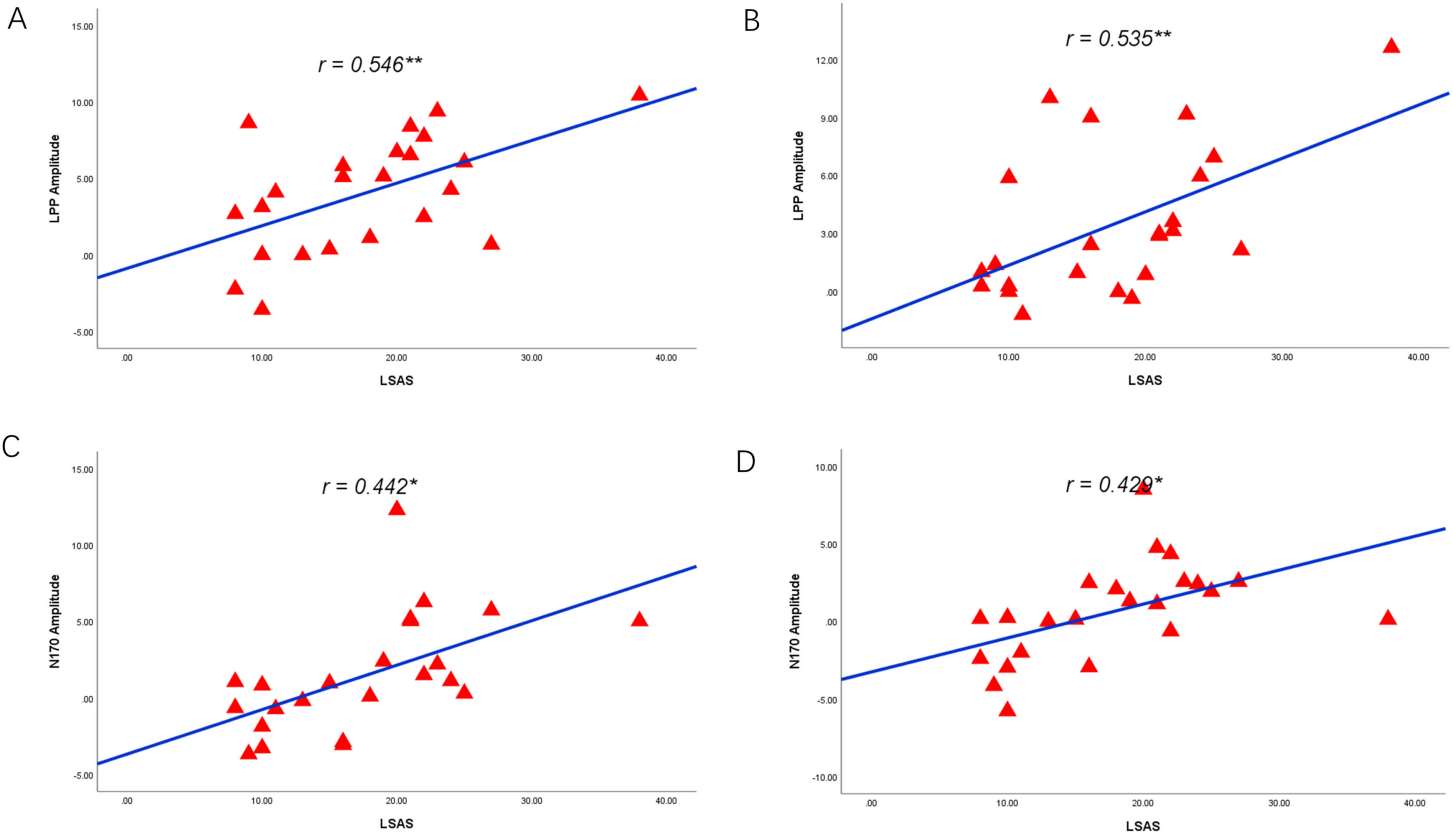
Correlations between reduced LPP, N170 and social anxiety symptoms. **(A)** Reduced LPP amplitude as a function of reduced LSAS scores under the attention sensitivity conditions. **(B)** Reduced LPP amplitude as a function of reduced LSAS scores under the attention disengagement conditions. **(C)** Reduced N170 amplitude as a function of reduced LSAS scores under the attention sensitivity conditions. **(D)** Reduced N170 amplitude as a function of reduced LSAS scores under the attention disengagement conditions. * 0.3<r<0.5, ** 0.5<r<1.0.

The ERP amplitude and LSAS scores were correlated, and this correlation analysis showed that the experimental group’s changes in social anxiety symptoms were associated with the amplitude of N170 and LPP from pretest to posttest. Specifically, reductions in social anxiety, as measured by LSAS scores, were found to be positively correlated with changes in LPP amplitude under the attention sensitivity conditions (*r* = 0.546**, *P* = 0.007) (see **Figure 13A**) and the attention disengagement conditions (*r* = 0.535**, *P* = 0.009) (see **Figure 13B**). Additionally, under the attention sensitivity conditions, a significant correlation was observed between the changes in N170 and the LSAS scores before and after treatment (*r* = 0.442*, *P* = 0.035) (see **Figure 13C**) and the attention disengagement conditions (*r*=0.429*, *P* = 0.041) (see **Figure 13D**).

## 4 Discussion

From a behavioral perspective, it was observed that hypnotherapy significantly reduced the attentional bias of individuals with social anxiety disorder (SAD), as evidenced by reduced attentional sensitivity to threatening stimuli and improved attentional disengagement difficulties, as well as significant improvement in social anxiety. The ERP results showed a distinction between the two groups. Specifically, the experimental group exhibited lower N170 amplitude at the posttest stage compared to the pretest stage. For LPP, the experimental group showed a significant decrease in amplitude at the posttest stage. However, we did not observe this change in the control group. Notably, the symptom improvements in the experimental group were positively correlated with their reduced N170 and LPP amplitude.

Meta-analyses have revealed the existence of attentional bias towards threat among anxious populations (47). The odd-one-out experimental paradigm used in this study extends previous studies based on the dot probe paradigm (48), the Stroop paradigm (49), and the cue-targeting paradigm (15). Importantly, the current study revealed that hypnotherapy might effectively modify the attention bias of socially anxious individuals. After the hypnosis intervention, there were significant improvements in both attentional sensitivity and attentional disengagement difficulties to angry faces. Our research indicated that hypnotherapy significantly reduced social anxiety scores. The results of the follow-up after one month showed that the effect of the hypnosis intervention persisted.

N170, a key neurophysiological marker of human face processing, largely accounts for the cognitive neural mechanisms of face expression processing (50, 51). Previous studies have shown that the high social anxiety group exhibited significantly enhanced N170 amplitude in comparison to the low social anxiety group (52). The findings of the present study showed that after the hypnosis intervention, the N170 amplitude elicited by emotional faces was significantly lower in the experimental group, suggesting an improvement in face recognition. Participants who received hypnotherapy had lower N170 amplitudes for angry faces after hypnosis. This pattern was not found for happy faces. Our study found that N170 can be reduced through a treatment designed to improve attention bias and gradually desensitize individuals to scary scenarios. These findings not only contribute to the literature on neural markers of treatment response in social anxiety but also demonstrate the effectiveness of hypnosis intervention.

In general, there is a noticeable influence that angry faces have on early attention allocation. However, the benefits of hypnotherapy intervention for people with SAD are not only due to the above-mentioned early processing efficiency. LPP is a classic neural marker for facilitated attention to emotional stimuli, and its decrease indicates the achievement of successful voluntary emotional regulation (53–55). Research has shown that LPP has greater modulation in response to unpleasant stimuli (56, 57). At the posttest stage, we found that the experimental group’s LPP amplitude decreased when they performed the odd-one-out task, suggesting that hypnosis changed their attentional sensitivity to emotional faces and difficulty disengaging from angry faces.

N170, representing early bottom-up processing (58), and LPP, representing late top-down processing (59), are likely involved in how hypnosis modifies attentional bias in SAD. The decreased N170 amplitude following hypnotic intervention showed that SAD participants had reduced attentional sensitivity and vulnerability, and they were more likely to disengage from negative emotional faces. In contrast, reduced LPP amplitude indicated the inhibition of top-down processing sensitivity and disengagement difficulties to negative emotional faces. In this case, fewer cognitive resources were required to modulate negative emotions, which was favorable to top-down processing, and the LPP amplitude was reduced (60). The present study has revealed that hypnosis can modulate not only late processing but also early processing, despite early processing previously being thought to be more difficult to modulate (61).

Interestingly, the correlation between reduced LPP and N170 amplitudes and social anxiety symptoms, as indexed by LSAS scores, may offer more proof of the relationship between the two components and symptoms of SAD. The neural generator of N170 elicited by schematic faces is the fusiform gyrus, which is responsible for the holistic processing of a face (62), while the LPP reflects more of the activity of occipital, inferotemporal, and parietal regions involved in emotion processing (63, 64). These results indicate that changes in face processing and attention towards emotional stimuli, as indexed by N170 and LPP, may be the principal ways in which hypnotherapy intervention produces effects. The attentional bias of people with SAD was successfully changed; thus, hypnotherapy can improve the symptoms of social anxiety and change attentional bias at the same time.

This study used hypnosis to integrate the progressive relaxation technique of systematic desensitization therapy, using hypnotic suggestions to keep the participants relaxed in social anxiety situations and to produce a positive and pleasant experience. This hypnosis technique can both desensitize the subject to anxiety in a relaxed state and associate the anxious situation with a pleasant experience (producing positive reinforcement) to form a new conditioned reflex to the anxious situation, thus effectively improving social anxiety. With the help of ERPs, which have been used in research on the hypnotic brain mechanisms, we discovered that hypnotic alteration of social anxiety is accompanied by measurable changes in N170 and LPP amplitude. These findings suggest that the mechanisms by which hypnotherapy produces clinical effects may be changes in attention bias towards angry faces, as measured by N170 and LPP. These findings, including one of our previous studies (25), suggest that attentional bias may be a focused target for the treatment of social anxiety. Hypnotherapy, identified as an innovative therapeutic approach, has shown remarkable efficacy in mitigating SAD. Nonetheless, when compared to CBT, the long-term efficacy and relapse rates of hypnotherapy necessitate additional research. CBT, recognized as one of the standard interventions for SAD, engages in identifying and modifying patients’ cognitive schemas, incorporates behavioral experiments and homework assignments, and aids patients in progressively confronting social contexts, diminishing anxiety levels (65). The short-term efficacy of CBT is well-supported by extensive empirical research, exhibiting favorable long-term outcomes and minimal relapse rates (66–68). In clinical practice, hypnotherapy can act as a supplementary treatment, presenting an alternative for patients with suboptimal or partial responses to conventional treatments. Moreover, the administration of hypnotherapy mandates that practitioners possess specialized hypnotic expertise and experience. CBT has emerged as a frontline treatment for social anxiety disorder, extensively adopted in clinical contexts and underpinned by robust empirical research. Doctors need to carefully weigh patients’ individual conditions, therapeutic requirements, and the attributes and suitability of diverse treatment methods when devising treatment plans. Tailored therapeutic strategies and integrated treatment approaches can more effectively address patient needs, enhance treatment outcomes, and improve patients’ quality of life.

Previous studies on hypnotherapy for SAD have been limited to case reports and small analogue studies, making this study an innovation that enriches intervention methods for social anxiety. However, there are still limitations in this study: (1) objective physiological indicators of social anxiety were not assessed, and future studies should detect changes in subjects’ heart rate, respiration, blood pressure, and blood oxygen levels before and after the intervention; (2) the present sample size for ERP experiments is notably small, which may compromise the stability and reliability of the findings. To address this issue, future research should consider increasing the sample size, potentially through enlarging the subject pool, extending the observation period, or employing more extensive recruitment strategies and (3) the present study only assessed attentional bias from the N170 and LPP components of event-related potentials, and future studies could combine other technical means, such as functional magnetic resonance imaging, to further clarify the neural mechanism of hypnosis for social anxiety and identify the mechanism of the intervention effect of hypnotherapy to apply this technique to the practice of social anxiety population intervention.

## 5 Conclusion

This study investigates the effects of hypnosis on the behavior and electrophysiology of individuals with SAD. The present study demonstrates that hypnotherapy can improve the symptoms of social anxiety in individuals with SAD. Meanwhile, hypnosis intervention reduced the amplitude of the N170 and LPP components, which altered their attention to become less sensitive to threatening information. These findings suggest that hypnosis can be an effective method to reduce SAD, and N170 and LPP could be considered as potential biomarkers for its treatment.

## Data Availability

The raw data supporting the conclusions of this article will be made available by the authors, without undue reservation.
