# Supplementary material for: Hypnotherapy modulating early and late event-related potentials components of face processing in social anxiety
Source: Front Psychiatry. 2024 Oct 16;15:1449946. doi: 10.3389/fpsyt.2024.1449946 (PMC11528304; doi:10.3389/fpsyt.2024.1449946)
Supplement: Supplementary file 1 [file Table1.docx]

Supplementary Material

**Hypnotic Suggestions**

Now, enjoy this wonderful feeling.

Please relax (pause for 5 seconds). deeply relax (pause for 5 seconds). continue to stay in the hypnotic state.

Now, imagine that you are in a public place which is very crowded. You are eating and drinking (pause for 5 seconds). You feel very relaxed and comfortable (pause for 5 seconds). It reminds you of a good time in the past (pause for 5 seconds).

Now, imagine you are meeting a stranger. You are sitting face-to-face with the stranger (5-second pause). You feel very relaxed, very comfortable (5-second pause). It makes you feel as good as if you are sitting with someone you really like. It’s a wonderful, happy time (5-second pause).

Now, imagine yourself attending a group activity where you are the main character and the center of attention (pause for 5 seconds). It's very easy and comfortable for you (5 second pause). You are like a famous facilitator, leading the group in a fun game with ease (pause for 5 seconds).

Imagine you are attending an important meeting and you are going to speak at it… (pause for 5 seconds) You walk up to the podium very relaxed and at ease (pause for 5 seconds). You feel like a famous orator, delivering a stirring speech with a lot of eloquence and ease (pause for 5 seconds).

Now, please experience this very relaxed and happy mood when you interact with people (pause for 5 seconds). Please experience the wonderful and accomplished feeling of being the center of attention in public and being noticed by everyone (pause for 5 seconds).

When you wake up, you can still experience a very relaxed and enjoyable feeling when interacting with people (pause for 5 seconds). You will be more confident and relaxed to talk and chat with others (pause for 5 seconds).

You greatly enjoy interacting with strangers (pause for 5 seconds). You greatly enjoy interacting with important and authoritative figures (pause for 5 seconds). You greatly enjoy standing in the center of the stage and being watched by everyone (pause for 5 seconds).

You yearn to interact with all sorts of characters, regardless of gender or status (pause for 5 seconds). You like to interact with all sorts of characters, even if you see an angry face, an angry face, you will be very calm (pause for 5 seconds), because you like to interact with people, (pause for 5 seconds), and others like to interact with you (pause for 5 seconds).

You get along well with everyone you meet (pause for 5 seconds). You are full of energy every day (pause for 5 seconds). You like yourself (pause for 5 seconds). You are unique. You are the best. You are a very charming person. You are a unique person. You are a perfect person (pause for 5 seconds).

You are a very capable person (pause for 5 seconds). No matter what happens you can handle it easily (pause for 5 seconds). when faced with a difficult problem, you can easily find a solution (pause for 5 seconds). You are the master of your own fate. You strongly believe that success does not rely on luck, but on ability and effort (pause for 5 seconds).

You are very capable of shaping things. you are the master of your destiny (pause for 5 seconds).

Okay, you are now very relaxed (pause for 5 seconds). You are still asleep (pause for 5 seconds). You feel very relaxed, relaxed all over (pause for 5 seconds).

Please stay completely relaxed (pause for 5 seconds). Concentrate on what I am about to say to you (pause for 5 seconds).
